# Embolic Phenomena of Libman-Sacks Endocarditis and Antiphospholipid Syndrome

**DOI:** 10.7759/cureus.46957

**Published:** 2023-10-13

**Authors:** Asher Gorantla, Michael Schaible, Shruthi S Sivakumar, Anandita Kishore, Wayne Andrew-Palmer, Selin Unal, Michael Ramirez, Varshitha Panduranga, Adam S Budzikowski

**Affiliations:** 1 Cardiology, State University of New York (SUNY) Downstate HSC, Brooklyn, USA; 2 Internal Medicine, State University of New York Downstate Health Sciences University, Brooklyn, USA; 3 Neurology, State University of New York Downstate Health Sciences University, Brooklyn, USA; 4 Internal Medicine, Sisters of Charity Hospital, Buffalo, USA; 5 Division of Cardiovascular Medicine – Electrophysiology section, State University of New York Downstate Health Sciences University, Brooklyn, USA

**Keywords:** : ischemic stroke, embolic phenomena, systemic lupus erythromatosus, libman sacks endocarditis (lse), antiphospholipid antibody

## Abstract

Patients with systemic lupus erythematosus (SLE) and antiphospholipid antibody syndrome (APS) are at high risk of developing arterial or venous thromboembolism and a state of systemic hypercoagulability. Libman-Sacks endocarditis (LSE) is a type of non-bacterial endocarditis usually seen in patients with systemic lupus erythematosus and antiphospholipid antibody syndrome. These vegetations dislodge easily and can cause profound neurological and systemic complications in the form of emboli. We describe one such case of a young woman with known SLE who presented with an acute middle cerebral artery (MCA) stroke and was found to have APS with extensive mitral valve vegetation, indicating Libman-Sacks endocarditis on echocardiography. Recognizing the increasing frequency of both APS and LSE in patients with SLE and screening patients, especially the younger population with SLE, for APS is vital. Furthermore, in those patients presenting with embolic events, echocardiography plays a key role as it can help expedite the diagnosis of LSE. Our case report also reiterates that warfarin, when compared to direct oral anticoagulants (DOAC), is superior in decreasing future embolic events.

## Introduction

Systemic lupus erythematosus (SLE) patients with antiphospholipid syndrome (APS) have a higher prevalence of arterial or venous thromboembolism as well as intracardiac valvular involvement in the form of Libman-Sacks endocarditis (LSE) [1-3]. Cerebral ischemia is the most common arterial manifestation of APS, accounting for up to 50% of all events [4], and the presence of LSE increases the risk of cerebromicroemboli by three times per hour [5,6]. We describe one such case of a young woman with known SLE who presented with an acute middle cerebral artery (MCA) stroke and was found to have APS with extensive mitral valve vegetation, indicating Libman-Sacks endocarditis on echocardiography.

## Case presentation

**Figure 1:**
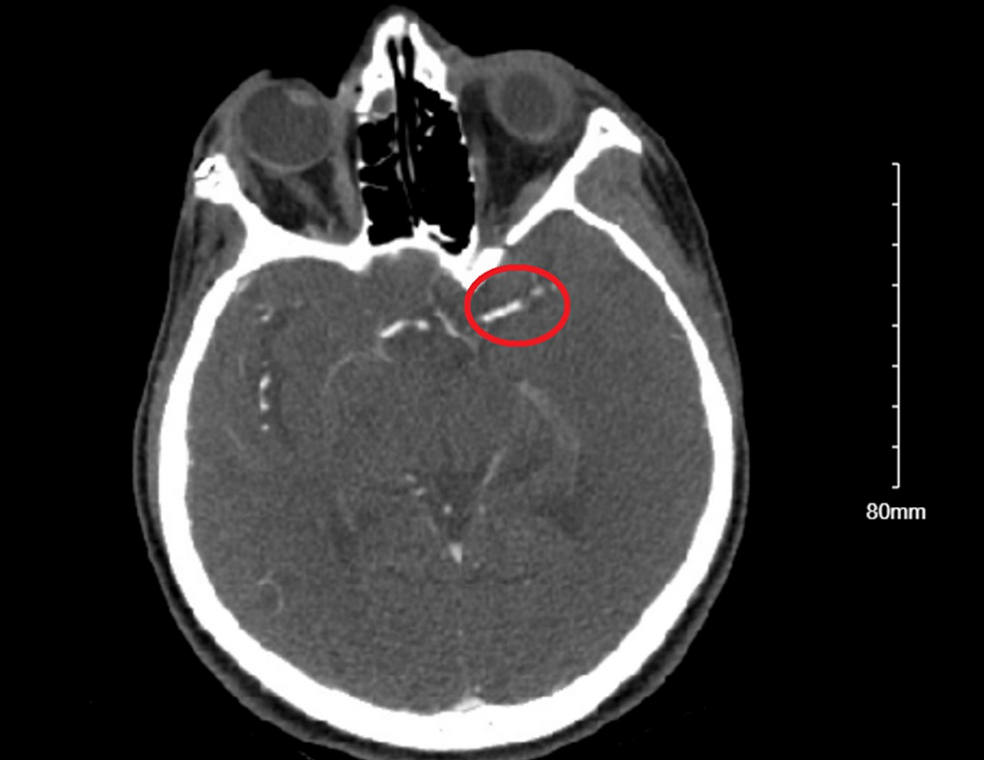
Computed tomography angiography showing occlusion of the left M1 division of the middle cerebral artery.

A 23-year-old woman with systemic lupus erythematosus, pulmonary embolism, and deep vein thrombosis on apixaban with good compliance presented with acute-onset aphasia and right face, arm, and leg weakness. A physical exam showed global aphasia, right facial, arm, and leg weakness, as well as left gaze preference. Computed tomography of the head showed an established, large acute infarct in the left MCA territory, with a computed tomography angiogram showing acute occlusion of the proximal left MCA (Figure 1 and Video 1).

**Video 1:** Magnetic resonance imaging showing a large territory of diffusion restriction in left middle cerebral artery region

**Figure 2:**
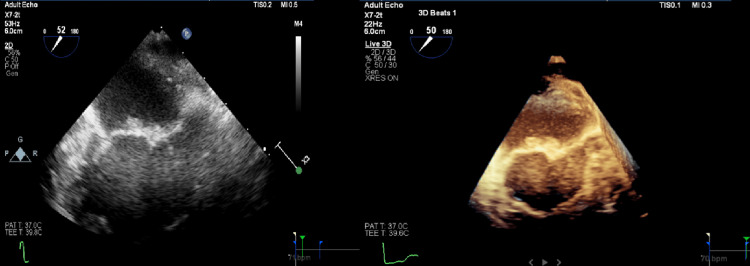
Transesophageal echocardiography demonstrating multiple large vegetations on the anterior and posterior leaflets of the mitral valve.

Thrombolysis and thrombectomy were deferred due to the time of presentation and size of the infarct. Further workup included blood cultures for infective endocarditis and hypercoagulable workup for Factor V Leiden, protein C, S deficiency, and antithrombin III deficiency, which were all negative, and antibodies for APS. Serological testing revealed positive beta-2-glycoprotein, anti-cardiolipin antibodies, and lupus anticoagulant antibodies. Transesophageal echocardiography was done to rule out cardiac etiology, and it showed multiple large vegetations on the anterior and posterior leaflets of the mitral valve, indicative of Libman-Sacks endocarditis (Figure 2).

The patient’s anticoagulation was transitioned from apixaban to warfarin, as is the standard of care for the treatment of antiphospholipid antibody syndrome. The patient was monitored, and her symptoms improved with physical therapy. Repeat testing for APS antibodies after 12 weeks was persistently positive for beta-2-glycoprotein, anti-cardiolipin antibodies, and lupus anticoagulant antibodies. The patient was followed up with scheduled outpatient visits thereon, and no further embolic events were noted.

## Discussion

Antiphospholipid antibody syndrome is a multisystemic autoimmune disorder characterized by the presence of antibodies against phospholipid-binding proteins in the setting of hypercoagulability and arterial and venous thrombosis. APS may be associated with other autoimmune conditions, has been estimated to be present in up to 30% of patients with SLE, and is usually more severe in this context, with a higher incidence of thromboembolic events. Morbidity is high in APLS, with more than 30% of patients developing severe disability within 10 years [3].

Libman Sacks endocarditis, also known as non-bacterial thrombotic endocarditis or marantic endocarditis, develops as a result of endothelial injury against the background of a hypercoagulable state and most commonly involves the mitral valve. It is seen in up to 11% of patients with SLE and has been observed in approximately 32-38% of patients with APS [3]. Delayed detection in at-risk patients can lead to destructive cerebrovascular events, peripheral arterial embolic events, the development of infective endocarditis, and an overall increase in morbidity and mortality.

Antiphospholipid antibody syndrome independently promotes the formation of valvular thrombi. In addition, the antiphospholipid antibodies themselves contribute to endocarditis, as was confirmed by the presence of subendothelial immunoglobulin deposition of anti-cardiolipin antibodies within these vegetations [4]. As was seen in our patient, the presence of Libman-Sacks endocarditis is associated with a higher risk for embolic cerebrovascular disease [5].

**Figure 3:**
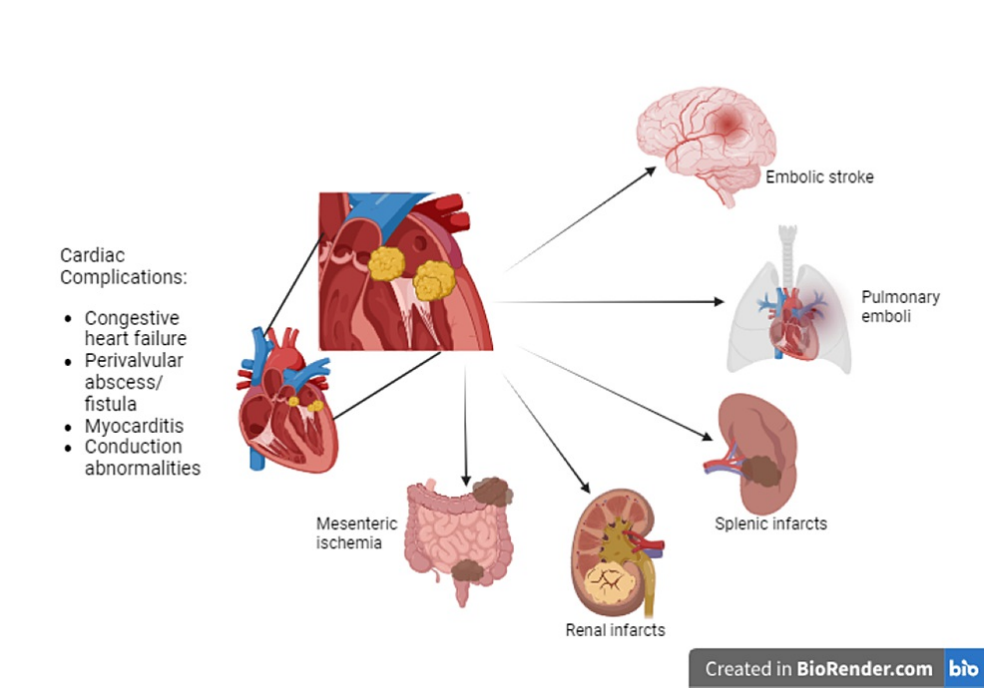
Systemic manifestations of Libman-Sacks endocarditis Image credit: Asher Gorantla

Libman-Sacks vegetations are more easily dislodged when compared to infective endocarditis systemically due to very little inflammatory reaction at the attachment site at the valve. Microscopically, these lesions are characterized by fibrin deposits at various stages of fibroblastic organization and neovascularization and by a variable extent of inflammation, with immune complexes, mononuclear cells, and thrombi interwoven with fibrin strands. Patients can develop extensive embolic manifestations such as cerebral emboli, mesenteric ischemia, pulmonary embolism, and splenic and renal infarcts [4] (Figure 3).

Trans-esophageal echocardiography is preferred for the detection of LSE over transthoracic echocardiography. These vegetations are characterized by the presence of irregular borders and heterogeneous echo density and usually do not display independent motion. The basal and mid-portion of the mitral and aortic valves are involved most frequently. Diffuse or focal leaflet thickening of the mitral and aortic valves is observable. The involved valves may exhibit regurgitation. Studies have demonstrated that, in addition to the higher sensitivity of TEE for the detection of these lesions, TEE was also more positive for the specific localization, size, and extent of these vegetations [6,7]. Although a biopsy of the vegetation is the definitive diagnosis, this may not always be feasible. Alternatively, serological testing, transesophageal echocardiology, clinical findings, negative blood cultures, and no response to antibiotic treatment are markers that invariably help to confirm the diagnosis of LSE.

This case illustrates the importance of screening SLE patients for APS due to the devastating consequences of untreated disease. Antiphospholipid antibodies are associated with approximately 100,000 strokes and myocardial infarctions annually [8,9]. Catastrophic APS is the most severe and potentially fatal form of APS, developing from multi-organ failure in the setting of extensive systemic microthrombi formation [1]. Patients with APS can present with other systemic manifestations, which have been depicted in Figure 3. As in the patient discussed in our case, NBTE is not typically screened for unless a new heart murmur is detected on a physical exam, a cardioembolic stroke occurs, or new-onset heart failure develops.

Diagnosis of antiphospholipid antibody syndrome

The European League Against Rheumatism 2019 guidelines recommend that every patient diagnosed with SLE should be tested for APS, which is diagnosed by one or more unexplained thrombotic events or adverse obstetric outcomes in the presence of persistently elevated anticoagulant antibodies. Baseline anti-ꞵ2 glycoprotein antibodies, lupus anticoagulant, and anticardiolipin antibodies should be obtained, and if anyone is positive, they should be repeated in 12 weeks. Two positive tests increase the likelihood of APS [10,11]. A retrospective study conducted on 204 patients with APS found that patients who were triple antibody positive (33%) were associated with a higher incidence of initial or recurrent thrombosis [12]. APS can be divided into primary APS and secondary APS. Secondary APS occurs in the presence of an underlying autoimmune condition or malignancy or may be drug-induced. Primary APS is defined as APS that occurs in the absence of these conditions.

Management of antiphospholipid antibody syndrome

Treatment options aim to lower serologic hypercoagulability or antiphospholipid antibody levels with immunosuppression. Immunosuppression is indicated only in cases of secondary APS with an underlying autoimmune etiology.

The role of anticoagulation in APS can be divided into primary prevention and secondary prevention. Further, for primary and secondary prevention, the disease may be stratified based on antibody titer levels into low, medium, and high-risk APS as per the 2019 European League Against Rheumatism guidelines. Primary prevention applies to those patients who have not had APS-related thrombosis or those with mildly elevated antiphospholipid antibodies. For patients who, by definition, have a high-risk type of APS (persistently high antibody titers or triple positivity), low-dose aspirin at 81 mg is advised [13].

Secondary prevention refers to patients with positive APS testing who have been recently treated for a thrombotic event. Most nonpregnant individuals with APS-related thrombosis require lifelong anticoagulation with warfarin [14,15]. Treatment with warfarin with a goal internal normalized ratio (INR) of 2-3 is the gold standard in patients with APS.

Five randomized controlled trials (RCT) analyzing 648 patients with APS divided into direct oral anticoagulant (DOAC) and warfarin treatment arms showed an increased incidence of arterial thrombosis in DOAC-treated patients than in warfarin-treated patients (OR = 5.168, 95% CI = 1.567-17.04, p = 0.007) [16]. A meta-analysis of 472 participants in four open-label RCTs performed by Khairani et al. demonstrated that overall, the use of direct oral anticoagulants compared to vitamin K antagonists was associated with increased odds of arterial thrombotic events, with no major difference between venous thromboembolism or major bleeding (OR: 5.43; 95% CI: 1.87-15.75; P < 0.001, I2 = 0%) [17]. In another RCT performed among a small cohort of 48 patients with APS, 6 out of 23 patients developed strokes on apixaban, compared to 0 out of 25 in the warfarin treatment arm [18].

Patients who have recurrent thrombotic events despite therapeutic INR levels and compliance can be considered to add low-dose aspirin, increase the INR goal to 3-4, or switch to low-molecular-weight heparin. There is insufficient evidence to determine the efficacy and safety profile of this individualized regimen.

Patients with valvular deposits started on anticoagulation do not appear to decrease the size of deposits but may prevent further deposition and may decrease future embolic events [19]. Lastly, one can consider surgical consultation if a patient develops recurrent thromboembolic events despite appropriate anticoagulation, or in severe mitral regurgitation or large vegetation [20].

## Conclusions

Recognizing the increasing frequency of both APS and LSE in patients with SLE and screening patients, especially the younger population with SLE, for APS is vital. Furthermore, in those patients presenting with embolic events, echocardiography plays a key role as it can help expedite the diagnosis of LSE. Our case report also reiterates that warfarin, when compared to direct oral anticoagulants, is superior in decreasing future embolic events.
